# The effects of entrepreneurial narrative on entrepreneurial intention: An affective events perspective

**DOI:** 10.1371/journal.pone.0304906

**Published:** 2024-09-06

**Authors:** Zhao Dong, Mingxu Bao

**Affiliations:** 1 School of Management and Science and Information Engineering, Jilin Business Big Data Research Center, Jilin University of Finance and Economics, Changchun, China; 2 School of International Business, Jilin International Studies University, Changchun, China; West Pomeranian University of Technology, POLAND

## Abstract

Entrepreneurial narrative has been used in the entrepreneurship education process to develop students’ entrepreneurial intention. However, previous research has less knowledge about the mechanisms between entrepreneurial narrative and entrepreneurial intention from an affective events perspective. We explore the effect of entrepreneurial narrative on entrepreneurial intention based on the theory of affective events theory. The sample of this study comprised 348 individuals who participated in entrepreneurship education in China. The results suggest that entrepreneurial narrative have a significant positive impact on college students’ entrepreneurial intention. Additionally, the relationship between entrepreneurial narrative and entrepreneurial intention is mediated by entrepreneurial passion, and entrepreneurial support positively moderates the relationship between entrepreneurial passion and entrepreneurial intention.

## Introduction

Entrepreneurship among college students is a crucial strategic direction for driving national innovation and promoting social and economic development [1]. College students, as an important part of the youth group, have the characteristics of vibrant and proactive, and thus they are more likely to be inspired by entrepreneurial stories and participate in activities [2,3]. Entrepreneurship education has emerged as a crucial strategy for fostering entrepreneurial activities among college students in China [4,5]. Existing literature has contributed to understanding specific aspects of entrepreneurship education, such as cultivating entrepreneurial abilities [6–8], enhancing entrepreneurial perception [9–12], develop positive emotions [13], and fostering entrepreneurial intention [14–18]. Despite literature on entrepreneurship education emerging, there is still confusion about what effective means and tools can be used in the entrepreneurship education process to achieve the desired educational goals [16].

Scholars have suggested that entrepreneurial narrative has the ability to contextualize entrepreneurship and can be an important pedagogical means for entrepreneurship education [19]. Indeed, there is some empirical evidence that entrepreneurial narrative has a positive effect on the formation of entrepreneurial identity [20,21], the conceptualization of entrepreneurial futures [22,23], the construction of entrepreneurial meanings [24], and the formation of entrepreneurial intention [24,25]. Morris et al.(2012) also proposes that entrepreneurship can be conceptualized as a series of discrete entrepreneurial events [26]. Entrepreneurial narrative is creative portrayals of entrepreneurial stories and events through language [24]. These stories and events have the potential to influence an individual’s perceptions, intentions, and decisions about the entrepreneurial process [25]. However, in entrepreneurship education research, the limited research about entrepreneurial narrative primarily has been centered on linguistic elements, with little attention paid to affective events as a critical factor. In this study, we follow this line of reasoning and present a theoretical framework to investigate the mechanism of the relationship between entrepreneurial narrative and entrepreneurial intention.

Entrepreneurial passion is an intense positive emotion toward entrepreneurial tasks and activities important to the entrepreneurial intention [27]. Scholars have argued that the intense positive emotion associated with entrepreneurial passion has initiative, and that it manifests itself in working hard on entrepreneurial intention [28]. According to affective events theory, individuals’ attitudes can be influenced by positive or negative events that they perceive, through their emotional responses [29]. The telling of entrepreneurial events through entrepreneurial narrative is often accompanied by strong entrepreneurial passion [30,31]. Individuals can deeply recognize and understand the passionate elements in these stories, which generates positive emotional involvement [32,33], stimulates strong entrepreneurial passion, and enhances entrepreneurial intention [34]. To explain the mechanisms underlying the relationship between entrepreneurial narrative and entrepreneurial intention, we draw on theories of affective events theory to argue that narrative leads to making passion, which results entrepreneurial intention.

We draw, too, on affective events theory, which states that changes in the external environment affect the process of changing individuals’ attitudes [29]. Entrepreneurship is the process of interaction between individuals and their environment, and individuals tend to respond favorably to attitudes when they are motivated by environmental support [35,36]. Entrepreneurial support includes university and government entrepreneurship courses, special funds, business incubators, and other assistance [37]. In this case of support, high passion can lead to an increase in entrepreneurial intention [38]. However, the critical role of entrepreneurial support in affective events about entrepreneurship has not been fully studied yet. Therefore, we leverage entrepreneurial passion as a mediate variable and entrepreneurial support as a moderate variable to investigate the mechanisms linking entrepreneurial narrative and entrepreneurial intention.

To fill these research gaps, this study aims to answer the key questions. Q1: How entrepreneurial narrative inspires entrepreneurial intention based on affective events theory? Q2: what external condition influences the relationship between entrepreneurial narrative and entrepreneurial intention? To answer these two questions, this study is based on the questionnaire data of Chinese college students, and SPSS 22 and AMOS 21 software are used for regression analysis and empirical tests.

This study seeks to contribute to the literature in several ways. First, we explore the effect of entrepreneurial narrative on entrepreneurial intention from the perspective of affective events. Adopting such an affective events perspective appears to be particularly important in entrepreneurship because starting a new business is a dynamic process in which entrepreneurs experience repeated cycles of successes and setbacks events with different emotions [39,40]. Entrepreneurial narrative can bring emotional resonance to college students through lecturing on entrepreneurial stories and events, making them feel a strong sense of identity and stimulating a strong entrepreneurial intention. We extend the contexts of application of affective events theory in the entrepreneurship literature by introducing entrepreneurial narrative into entrepreneurship education research. Second, we examined the pivotal role of entrepreneurial passion and entrepreneurial support. Prior studies have discussed the role of linguistic elements of entrepreneurial narrative in relation to entrepreneurial resources from a linguistic perspective. Gartner (2007) has proposed that, the analytic and cognitive processes inherent to entrepreneurial narrative serve as the “fuel” that ignites passion [24]. Entrepreneurial passion is important because it may influence individuals’ creativity, attention, and decision-making [27,41]. Furthermore, research has shown that the variability in motivational and emotional variables is related with degree of environmental support [8,42]. Thus, we examined the entrepreneurial passion and entrepreneurial support as two important factors that help to explain why and under which conditions entrepreneurial narrative affects entrepreneurial intention.

This study is structured as follows: First, we discuss the relevant literature and present a theoretical model based on affective events theory. Second, we explore the mechanism of entrepreneurial narrative on entrepreneurial intention and formulate the main hypotheses to be tested. Third, we examine the mediating role of entrepreneurial passion in the relationship and the moderating effects of entrepreneurial support. Fourth, the empirical process and results are presented along with the discussion and the conclusion.

## Theory and hypotheses

### Affective events theory

Affective events theory examines the structure, triggers, and consequences of individuals’ emotional responses in the workplace [29]. It argues that positive or negative work events can trigger an individual’s emotional responses [43]. These emotional responses, in turn, influence the individual’s attitudes and behaviors. Positive affective events can not only have a positive affective impact but also effectively buffer negative emotions brought about by challenging events [44,45]. Additionally, it is important to note that not all events can elicit emotional reactions. Mild events that are not related to an individual’s goals and values may only be assessed in the initial evaluation stage, thus not inducing emotional reactions [46]. Affective events theory systematically reveals the affective mechanism of events on individuals. For example, an entrepreneurial remarkable event or inspiring story is likely to evoke positive emotions, such as passion, which may directly increase interest in entrepreneurial attitudes.

### Entrepreneurial narrative

Narrative, which originated in linguistics, refers to the creative re-description of the content and meaning of an activity [47]. Narrative is a way of knowing and understanding that allows us to capture the richness and diversity of the meanings of humanity [48]. Gartner (2007) conceptualized entrepreneurial narrative as the creative portrayal of the entrepreneurial story and events through language [24]. He explained the essence of entrepreneurial narrative lies in the contextualized re-enactment of entrepreneurial events, intending to explore and analyze the entrepreneur’s specific contextualized entrepreneurial activities in a specific context. Fellnhofer (2018) supports Gartner’s view that entrepreneurial narrative is the creative depiction of entrepreneurial stories by entrepreneurs to make entrepreneurial careers more attractive [49]. Martens et al. (2007) argue that entrepreneurial narrative is events that entrepreneurs tell, portray, and analyze about themselves or their businesses [50]. These narratives are important communication activities for raising capital [51–53], cultivating customers [54], and building legitimacy [55]. Ibarra and Barbulescu (2010) affirmed that entrepreneurial narrative can assist entrepreneurs in overcoming the challenges of information asymmetry and uncertain access to external resources by presenting information about the identity of the entrepreneurial enterprise [56]. Entrepreneurial narrative implies information, experiences, theories, and visions from the perspectives of entrepreneurial process [24]. Thus, entrepreneurial narrative is the activity in which narrators contextualize the entrepreneurial process through the interpretation of entrepreneurial events that demonstrate their knowledge and experience of the entrepreneurial process.

### Theoretical framework

We develop a theoretical model about the effect of entrepreneurial narrative on entrepreneurial intention.

First, entrepreneurship education is a systematic and enduring process. It is crucial to ensure that individual entrepreneurial willingness is essential for facilitating students’ future entrepreneurial activities [57]. Entrepreneurial narrative is an account in which the narrator describes and analyzes the process of entrepreneurship and significant events. Entrepreneurial stories and events serve are conveying information about entrepreneurs’ experiences, ideas, perceptions, understandings, and feelings [22]. The linguistic representation of these stories and events, whether narrated, depicted, played out, presented, or interpreted through oral or written forms, facilitates entrepreneurial willingness.

Second, entrepreneurial passion is an important internal condition. Affective events theory highlights the significance of examining emotional responses, as particular emotional responses are more likely to accurately predict specific intentions and behaviors [35,46]. Entrepreneurial narrative can evoke positive emotions that may awaken an individual’s perception of entrepreneurial identity, leading to entrepreneurial passion. Entrepreneurial passion is a long-lasting and intense emotion [22]. Enduring entrepreneurial intention may result from attitudinal changes due to the accumulation of positive emotional experiences over time and the formation of overall evaluative judgments after careful consideration [58,59]. Therefore, maintaining an individual’s passion for entrepreneurship is a crucial emotional component of entrepreneurial narrative that influence entrepreneurial intention.

Third, entrepreneurship support is an important external condition. Entrepreneurial intention may be influenced by emotional impulses triggered by entrepreneurial events [60]. However, for sustained and stable entrepreneurial intention, positive external conditions must be present [61]. Stronger support for entrepreneurship can assist individuals in overcoming resource constraints that are specific to their student status, expanding their personal information and social network sources, and safeguarding the process of developing passion and entrepreneurial identity. Thus, under the conditions of entrepreneurial support, entrepreneurial intention may be persistently stimulated.

**Fig 1:**
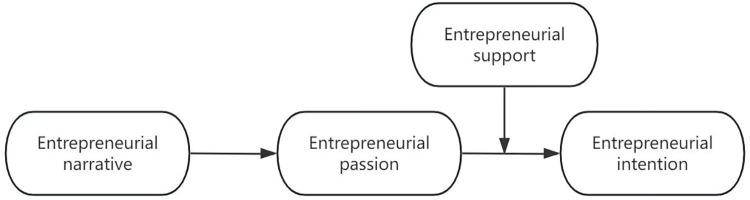
Theoretical framework.

Based on the above arguments, this study develops the following research framework in Fig 1.

### Entrepreneurial narrative and entrepreneurial intention

Entrepreneurial narrative is a means of creatively conveying information about entrepreneurship through storytelling [50]. The purpose of the narrative is to analyze the entrepreneur’s activity in a specific context, and the goal is to establish the entrepreneur’s identity and legitimacy [62,63]. The entrepreneurial narrative emphasizes that the narrator selects the narrative mode based on individual and situational differences. Successful narrators transform entrepreneurial processes and excellent entrepreneurial cases into inspiring stories through entrepreneurial narrative. They apply appropriate analytical methods to extract the implied entrepreneurial knowledge and revelations of entrepreneurial stories, promoting the circulation and transmission of concepts, ideas, and knowledge. This achieves an effect on the individual will, emotions, and behaviors of the recipients [30].

Entrepreneurial narrative aim to enhance the recipient’s cognitive understanding through verbal communication [50]. For college students, developing an entrepreneurial identity involves not only learning entrepreneurial knowledge systematically, but also gaining perception and accumulating entrepreneurial experience through observation, analysis, and reflection [64]. This process helps to identify key elements in the entrepreneurial process of successful entrepreneurs and learn ways to acquire resources, which can stimulate the entrepreneurial intention of college students [65]. The process of entrepreneurial narrative defines a new endeavor that can provide a positive explanation of creating value and help college students establish a sense of entrepreneurial identity [23]. College students’ identification with entrepreneurial identity will stimulate self-perception and experience under the effect of empathy. This, in turn, will enhance positive attitudes toward entrepreneurship, resulting in strong entrepreneurial intention. In summary, this paper proposes the following hypotheses:

H1: Entrepreneurial narrative has a significantly positive impact on entrepreneurial intention.

### The mediating effect of entrepreneurial passion

Entrepreneurial passion is a conscious positive emotion experienced by individuals engaged in activities that embody the characteristics of an entrepreneurial identity [27]. It involves intense positive feelings and identity centrality [66]. Positive emotions are pleasurable feelings that arise when an individual’s needs are satisfied after being stimulated by an internal or external event [35]. Entrepreneurial identity is generated when an individual learns about the characteristics of the entrepreneurial role and internalizes this role into their cognitive schema [66].

First, entrepreneurial narrative can promote entrepreneurial passion. On the one hand, narrative can stimulate individuals’ entrepreneurial positive feelings. Affective events theory suggests that cognitive appraisals of events are more capable of triggering affective responses than the events themselves [29]. Entrepreneurial narrative is the creative recounting, portrayal, and analysis of entrepreneurial stories and events by entrepreneurs. The stories constitute the content of the narrative, and the process of narrative gives meaning to the stories. Fellnhofer (2018) found that entrepreneurs can influence individuals’ perceptions, assessments, and decisions through narratives [49]. By analyzing entrepreneurial activities through the lenses of temporal and causal logic, entrepreneurial narrative attract individuals and vividly transmit entrepreneurial stories. This generates emotional resonance and positive entrepreneurial passion. On the other hand, entrepreneurial stories can help individuals identify with the role of an entrepreneur [67]. Entrepreneurial narrative can be used to analyze and evaluate entrepreneurial stories and experiences in-depth. This can guide individuals to form a concrete understanding of the entrepreneurial process and engage in relevant entrepreneurial activities. Entrepreneurial narrative can help individuals to acquire knowledge of the entrepreneurial process. It enables reflection on entrepreneurial activities and outcomes, leading to a conscious integration with the entrepreneurial story portrayed. This, in turn, fosters recognition and acceptance of the entrepreneurial identity.

Second, entrepreneurial passion can promote entrepreneurial intention. Entrepreneurial passion differs from other passions in that it is not only strong but also enduring. Individuals with entrepreneurial passion experience intense positive emotions that can more readily trigger positive judgments about a goal, thus promoting positive outcomes. Scholars have demonstrated that positive judgments are frequently accompanied by a greater degree of intention [68]. In other words, the conscious positive emotion embedded in entrepreneurial passion enhances the individual’s judgment of possible favorable outcomes in the future, thereby promoting the individual’s intention to engage in entrepreneurial activities. Furthermore, it has been observed that the majority of college students have not engaged in genuine entrepreneurial activities [69]. There is a need to cultivate a stronger sense of entrepreneurial identity among this demographic. Entrepreneurial passion can provide individuals with a meaningful identity connection of entrepreneur. This identity connection serves to enhance the sense of identification with entrepreneurship, thereby fostering a long-lasting intention to engage in entrepreneurial activities. Therefore, this internal drive inspired by entrepreneurial passion leads to a more lasting and stable entrepreneurial intention.

Third, entrepreneurial narrative can promote entrepreneurial intention through entrepreneurial passion. According to affective events theory, analyzing and evaluating an event that aligns with an individual’s goals and values can trigger an emotional response and lead to changes in attitudes and behaviors [35]. Scholars have noted that passion can significantly influence an individual’s willingness to engage in entrepreneurial activities [41]. Entrepreneurial narrative can offer detailed descriptions and analysis of entrepreneurs’ stories. They can enhance understanding of entrepreneurship principles and reignite their passion for this pursuit. Passion is an important and fascinating construct because it is often characterized as a “hot” feeling, or, metaphorically, “the fire of desire” [27,70]. Such a desire serves as a motivating factor for them to work towards their entrepreneurial goals and to overcome the difficulties and challenges of entrepreneurship [71]. The greater an individual’s entrepreneurial passion, the more confident they are in dealing with the challenges of the entrepreneurial process, which enhances their intention to engage in entrepreneurial activities. In summary, this paper proposes the following hypotheses:

H2: Entrepreneurial narrative has a positive impact on entrepreneurial passion.
H3: Entrepreneurial passion has a positive impact on entrepreneurial intention.
H4: Entrepreneurial passion mediates the relationship between entrepreneurial narrative and entrepreneurial intention.

### The moderating effect of entrepreneurial support

Entrepreneurial support refers to the government, university, and social funding available to support students in their entrepreneurial activities [37]. They provide entrepreneurial education, mentors, funding, and other support. It is important for entrepreneurial support in these institutions as the main venue for cultivating college students [38,72].

Effective entrepreneurial support can provide necessary and stable help, especially for college students. Entrepreneurial support reflects the recognition and supportive attitude of colleges and universities towards entrepreneurial behavior [73]. Colleges and universities can improve their students’ entrepreneurial knowledge, skills, and self-confidence by offering entrepreneurship courses, extracurricular competitions, and other practical activities [74]. This inspires and motivates students, enhances their recognition of entrepreneurial activities, and maintains their passion stable. Furthermore, during the planning and start-up process, new businesses frequently lack the necessary entrepreneurial resources [50]. Colleges and universities provide the necessary venues and financial support to carry out entrepreneurial activities. This helps college entrepreneurs overcome the resource constraints unique to their student status, expand their sources of information and social networks, and increase the possibility of entrepreneurial success. Students who receive entrepreneurial support are more likely to sustain their passion for entrepreneurship, which reinforces the effect of entrepreneurial passion on entrepreneurial intention. In summary, this paper proposes the following hypotheses:

H5: Entrepreneurial support moderates the positive relationship between entrepreneurial passion and entrepreneurial intention.

## Methodology

### Data collection

This study focuses on college students as the research subjects. A questionnaire survey was conducted on Chinese college students in Jilin, Liaoning, and Heilongjiang provinces. The questionnaire was designed according to the research model and the related scales were translated in both directions. The English literature questionnaires were translated into Chinese. Any questionnaires with noticeable differences were adjusted and modified. Three scholars from related research fields were invited to provide their objective opinions on potential issues with the questionnaire. Based on their feedback, the first draft of the questionnaire was revised. Thirty college students from Jilin Province were invited to participate and provide feedback on the questions. Based on their feedback, the questionnaire was revised and the final version was created. The pre-survey questionnaire was not included in the final sample.

We administered an anonymous questionnaire while fully respecting the data protection regulations through a survey link. From March to July 2022, 500 questionnaires were distributed and 396 were recovered, resulting in a recovery rate of 79.2%. After excluding incomplete and invalid questionnaires, 348 valid questionnaires were obtained. All interviewees were over 18 years old. They were briefed on the study’s aims, voluntary participation, and the right to withdraw. The informed consent form is provided in the questionnaire. Consent was obtained from all participants for inclusion in the study.

### Demographics

Table 1 provides the demographic characteristics of the sample. Respondents included 143 men (41.1%) and 205 women (58.9%). Of the total number of students, 60.1% were aged between 18 and 23, 18.1% were aged between 24 and 26, and 21.8% were aged over 26. The study included 62 students (17.8%) from the economic and management major, 74 students (21.3%) from the science and engineering major, 52 students (14.9%) from the medical major, 45 students (12.9%) from the art major, 32 students (9.2%) from the literature and history major, and 83 students (23.9%) from other majors.

**Table 1:** Demographic characteristics of respondents.

Characteristics	Category	Frequency	Percentage
**Gender**	Male	143	41.1%
	Female	205	58.9%
**Age**	18–23	209	60.1%
	24–26	63	18.1%
	>26	76	21.8%
**Major**	Economic and management	62	17.8%
	Science and engineering	74	21.3%
	Medical	52	14.9%
	Art	45	12.9%
	Literature and history	32	9.2%
	Other majors	83	23.9%

Note. N = 348.

### Variables and measures

#### Entrepreneurial narrative

We used 7 items to measure entrepreneurial narrative in our study. We based our measure of entrepreneurial narrative on items developed by Martens (2007) [50], and Lounsbury and Glynn (2001) [67]. The participants answered the items on a 7-point Likert scale anchored from “1 = strongly disagree” to “7 = strongly agree”. A sample item is “These entrepreneurial events and stories are very inspiring.” Cronbach’s α for this scale was estimated at 0.857.

#### Entrepreneurial passion

Entrepreneurial passion was measured using Cardon et al.’s (2013) 13-item scale [66]. Sample items include “It is exciting to figure out new ways to solve unmet market needs that can be commercialized” and “Establishing a new company excites me.” Responses were on a 7-point Likert-type scale, with 1 meaning “strongly disagree” and 7 signifying “strongly agree”. Cronbach’s α for this scale was estimated at 0.918.

#### Entrepreneurial support

We used 5 items to measure entrepreneurial support in our study. We based our measure of entrepreneurial support on items developed by Walter et al. (2013) [74]. Each respondent was asked to rate degree of support by using a 7-point Likert scale (1 = strongly disagree, 7 = strongly agree). A sample item is “The school can provide the necessary material support to initiate a business.” Cronbach’s α for this scale was estimated at 0.880.

#### Entrepreneurial intention

Entrepreneurial intention was measured using Liñán and Chen’s (2009) scale [75]. There are 6 items, with responses based on a 7-point Likert-type scale, ranging from 1 = strongly disagree to 7 = strongly agree. A sample item is “I am willing to work hard to become an entrepreneur.” Cronbach’s α for this scale was estimated at 0.846.

#### Control variables

We controlled for the respondent’s gender, age, and major at the individual level, as these factors may influence the relationships explored in the current study. Gender is the dummy variable, where 1 is male and 2 is female.

### Adequacy of the measures: Reliability, validity, and potential biases

In this study, SPSS 22 and AMOS 21 software were utilized to test the reliability and validity of all variables. Firstly, in terms of reliability, the reliability of the variables was tested by Cronbach’s α value and composite reliability (CR), and the Cronbach’s α value and CR value of all the variables were greater than 0.7, which indicated that the reliability of the scale was good. Secondly, in terms of validity testing, exploratory factor analysis (EFA) was performed on all question items of 348 valid questionnaires. The results revealed that the factor load value of each item above 0.6, and the average variance extracted (AVE) values above 0.5 are acceptable [76]. The square root of the AVE values of each variable was greater than the correlation coefficients with the other variables, indicating that the scale has good validity. In conclusion, it shows that this questionnaire has good reliability and validity. The results are presented in Table 2.

**Table 2:** Factor analysis and reliability analysis.

Variables		Items	Factor load	Cronbach’s α	AVE	CR
**Entrepreneurial Narrative**		EN1	0.726	0.857	0.508	0.878
		EN2	0.626			
		EN3	0.718			
		EN4	0.693			
		EN5	0.682			
		EN6	0.728			
		EN7	0.806			
**Entrepreneurial Passion**	**Intense Positive Feelings**	IPF1	0.785	0.964	0.733	0.965
		IPF2	0.761			
		IPF3	0.894			
		IPF4	0.866			
		IPF5	0.906			
		IPF6	0.843			
		IPF7	0.828			
		IPF8	0.878			
		IPF9	0.902			
		IPF10	0.885			
	**Identity Centrality**	IC1	0.857			
		IC2	0.884			
		IC3	0.776			
**Entrepreneurial Intention**		EI1	0.734	0.846	0.544	0.877
		EI2	0.705			
		EI3	0.729			
		EI4	0.795			
		EI5	0.731			
		EI6	0.730			
**Entrepreneurial Support**		ES1	0.798	0.880	0.644	0.900
		ES2	0.840			
		ES3	0.799			
		ES4	0.802			
		ES5	0.772			

Note. EN = Entrepreneurial Narrative; IPF = Intense Positive Feelings; IC = Identity Centrality; EI = Entrepreneurial Intention; ES = Entrepreneurial Support.

The Harman one-way test was utilized to analyze the homology bias of all valid questionnaires. The variance contribution rate of the first factor obtained without rotation was 29.425%, which was less than 40%, which indicates that the homologous bias problem of the research data was not severe and the data is reliable.

## Results

### Descriptive statistics and correlations

We use SPSS 22 statistical software to analyze the data. This study presents descriptive statistics and correlations of the variables. Table 3 shows the mean, standard deviation (SD), and correlation coefficient. Entrepreneurial narrative and entrepreneurial passion are significantly correlated with entrepreneurial intention. Entrepreneurial narrative is positively correlated with entrepreneurial passion. The results indicate that it is appropriate to examine the relationship between these variables in hypothesis testing.

**Table 3:** Mean, standard deviation, and correlation coefficient.

	Construct	Mean	SD	1	2	3	4	5	6	7
**1**	**Gender**	-	-	1						
**2**	**Age**	1.62	0.82	-0.332**	1					
**3**	**Major**	3.46	1.84	-0.100	-0.122*	1				
**4**	**Entrepreneurial narrative**	4.93	0.92	0.046	0.110*	0.034	1			
**5**	**Entrepreneurial passion**	5.33	0.89	0.157**	0.054	0.003	0.382**	1		
**6**	**Entrepreneurial intention**	5.35	0.93	0.015	0.173**	0.024	0.222**	0.253**	1	
**7**	**Entrepreneurial support**	4.80	0.99	0.063	-0.021	-0.007	0.287**	0.258**	0.164**	1

Note. N = 348. SD = standard deviation.

* p < 0.05

**p< 0.01.

### Analysis and results

**Fig 2:**
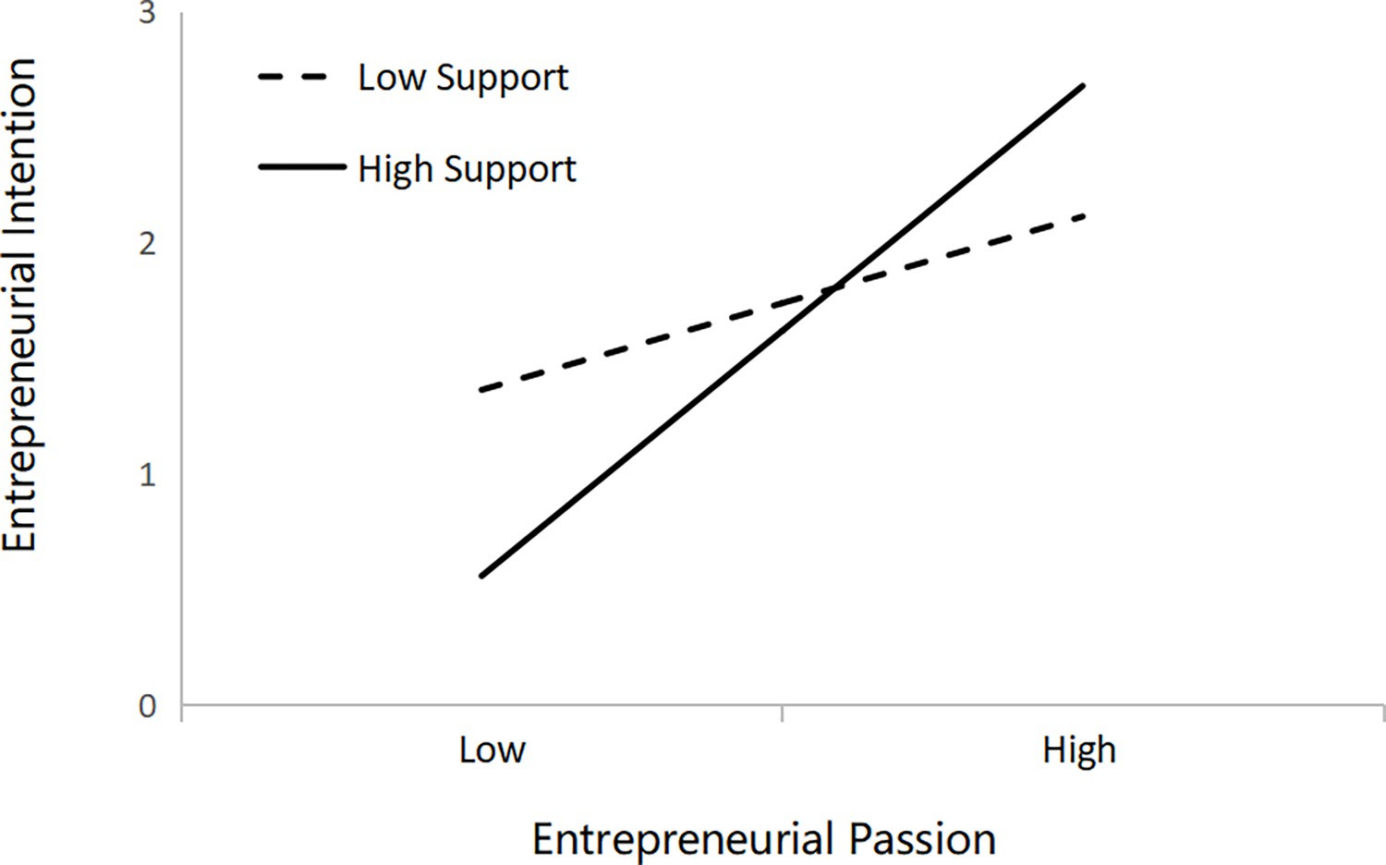
The moderating effect of entrepreneurial support on the link between entrepreneurial passion and entrepreneurial intention.

Step-by-step regression is applied to test the hypothesis, and the results are presented in Table 4. Gender, age, and major are control variables. Model 1 and Model 3 describe the relationship between control variables and dependent variables. Model 4 confirms a significant positive effect of entrepreneurial narrative on entrepreneurial intention (β = 0.198, p<0.001), verifying hypothesis H1. To test the mediating role of entrepreneurial passion, this study introduces the variable of entrepreneurial passion in Model 6, based on Model 4. Model 4 confirms a significant positive effect of entrepreneurial narrative on entrepreneurial intention (β = 0.198, p<0.001), verifying hypothesis H1. The results of Model 2 indicate that entrepreneurial narrative has a significant positive effect on entrepreneurial passion (β = 0.366, p<0.001), verifying hypothesis H2. In Model 6, entrepreneurial passion has a significant positive effect on entrepreneurial intention (β = 0.188, p<0.01), verifying hypothesis H3. Simultaneously, Model 4, Model 5, and Model 6 show that the addition of the variable of entrepreneurial passion decreases the facilitating effect of entrepreneurial narrative on entrepreneurial intention. The regression coefficient β decreases from 0.198 to 0.129, respectively, and the significance level decreases from p<0.001 to p<0.05. Therefore, it can be concluded that entrepreneurial passion plays a mediating role in the relationship between entrepreneurial narrative and entrepreneurial intention, thus verifying hypothesis H4. Models 5, 7, and 8 confirm that entrepreneurial support has a positive moderating effect on the relationship between entrepreneurial passion and entrepreneurial intention (β = 0.137, p<0.01). Therefore, hypothesis H5 is supported. Fig 2 portrays the moderating role of entrepreneurial support in the relationship between entrepreneurial passion and entrepreneurial intention.

**Table 4:** Results of regression analysis.

Variables	Entrepreneurial passion		Entrepreneurial intention					
	Model 1	Model 2	Model 3	Model 4	Model 5	Model 6	Model 7	Model 8
**Gender**	0.203***	0.165**	0.091	0.071	0.043	0.040	0.042	0.038
**Age**	0.126*	0.070	0.210***	0.180**	0.180**	0.167**	0.184**	0.189**
**Major**	0.039	0.016	0.059	0.047	0.050	0.044	0.051	0.053
**EN**		0.366***		0.198***		0.129*		
**EP**					0.236***	0.188**	0.207***	0.238***
**ES**							0.112*	0.113*
**EP × ES**								0.137**
**R** ^**2**^	0.039	0.170	0.039	0.077	0.093	0.107	0.104	0.122
**ΔR** ^**2**^	0.030	0.160	0.031	0.067	0.082	0.094	0.091	0.107
**F**	4.628**	17.521***	4.666**	7.187***	8.767***	8.176***	7.973***	7.906***

Note. N = 348. EN = Entrepreneurial narrative, EP = Entrepreneurial passion, ES = Entrepreneurial support, EP × ES = Entrepreneurial passion × Entrepreneurial support.

* p < 0.05

**p< 0.01

***p< 0.001.

## Discussion

This study developed a model of the role of entrepreneurial narrative on entrepreneurial intention and explored the influence mechanisms and boundary conditions. We utilize data from a survey of 348 individuals to analyze the impact of entrepreneurial narrative on entrepreneurial intention in China. Our findings contribute to research on improving the effectiveness of entrepreneurship education through entrepreneurial narrative.

First, the result shows that entrepreneurial narrative has a significant positive effect on entrepreneurial intention. This finding corroborates the findings that narratives have a positive impact on motivating individual attitudes and intentions [25,49,77]. The results of our empirical research also deal with the idea that entrepreneurial narrative can act as an important means and informational tool in the field of entrepreneurial education [25]. It helps individuals to recognize and understand the value of innovation and creativity and enhances their positive attitudes towards entrepreneurship, thereby leading to a strong entrepreneurial intention. This responded to the call for Zampetakis & Moustakis’s (2006) appeal, which asserts the necessity of investigating the factors that can influence an individual’s intention, particularly among the youth [78]. In addition, our findings complement research on entrepreneurship education outcomes in developing countries.

Second, using the conceptual model of Weiss and Cropanzano’s (1996) theory of affective events [29], we find that entrepreneurial passion acts as a mediator between entrepreneurial narrative and entrepreneurial intention. The applicability of affective events theory to entrepreneurial passion has received empirical support in the past [35]. Entrepreneurial narrative can offer a positive explanation for entrepreneurial outcomes, evoke entrepreneurial passion in the audience, and stimulate entrepreneurial intention. Our results indicate that entrepreneurial passion is not only a character to traditional entrepreneurs, but is a dynamic emotion that can be developed through entrepreneurship education [13]. This is also consistent with the findings in the field of entrepreneurship, which indicates that entrepreneurship is an emotional journey for both entrepreneurs and students [70,79]. In fact, by listening to the narratives of narrators, it becomes possible to begin to grasp the complex emotion of entrepreneurship [21]. This may increase important issues for the practice of stimulating entrepreneurial intention [25]. The finding of our study confirms that entrepreneurial passion could be a key point in stimulating entrepreneurial intention.

Third, entrepreneurial support plays a positive moderating role in the relationship between entrepreneurial passion and entrepreneurial intention. Our findings also reaffirm the research conclusion by Neneh (2022) [8]. Entrepreneurial support creates a positive external environment that is conducive to maintaining the stability of entrepreneurial passion, reducing external distractions [42], investing more energy and emotion [37], and reinforcing the impact of entrepreneurial passion on entrepreneurial intention. These results also present a more comprehensive understanding of the effect of support between entrepreneurial passion and entrepreneurial intention, as discussed by Maheshwari et al. (2023) [57]. Our results given by our empirical research enriched this line of research by exploring the impact of elements of narrative on entrepreneurial intention.

### Theoretical and practical contributions

#### Theoretical implications

Our findings have several theoretical implications for the literature on entrepreneurship education, emotion, and motivation. First, this paper expands the research on entrepreneurial narrative in entrepreneurship education. The existing literature on entrepreneurial narrative primarily focuses on how entrepreneurs tell stories based on capital stock [80]. However, it lacks an in-depth exploration of the deeper concepts of entrepreneurial narrative, such as cognition, emotion, and logic. Additionally, it fails to explore the impact of different characteristics of various entrepreneurial populations [56]. Entrepreneurial narrative is important for transferring information and stimulating emotions in entrepreneurship education. They can enhance college students’ entrepreneurial cognition, help them establish their identity as entrepreneurs, and promote positive attitudes toward entrepreneurship, ultimately leading to increased entrepreneurial intention. This paper expands the theoretical study of entrepreneurship education and students’ entrepreneurship by providing a new context for this study of entrepreneurial narrative.

Second, this paper examines how entrepreneurial narrative influence individuals’ entrepreneurial intention from an affective perspective. According to affective events theory, an individual’s emotional response and subsequent changes in attitude and behavior can be triggered by a positive analysis of an event [35]. Entrepreneurial narrative can help individuals learn entrepreneurial methods, stimulate their entrepreneurial passion, and promote their entrepreneurial intention. Narrative describing entrepreneurial experiences can evoke emotional resonance, stimulate entrepreneurial passion, and enhance willingness to engage in entrepreneurial activities [8]. Entrepreneurial narrative can convey an entrepreneurial spirit and evoke emotional resonance and involvement among college students. This is in line with the psychological characteristics of college students who are easily influenced and inspired by positive information in real-life situations [57]. This paper expands the research perspective on entrepreneurial passion and intention.

Third, this paper examines the moderating effect of entrepreneurial support on the relationship between entrepreneurial passion and entrepreneurial intention. Our study suggests that providing entrepreneurial support can offer more information and resources to entrepreneurs. Cardon et al. (2009) noted that a conducive environment is essential for the sustenance of an individual’s entrepreneurial passion [27]. Additionally, college students who have a passion for entrepreneurship tend to have higher confidence and recognition of entrepreneurial activities [8]. They may also perceive more entrepreneurial opportunities and be more willing to make entrepreneurial attempts, which can stimulate their entrepreneurial intention. This study’s results support the notion that the entrepreneurial activities of students arise from the interplay of personal factors and environmental conditions [42]. This study examines the impact of environmental and entrepreneurial affective factors on forming entrepreneurial intention. This sheds light on the psychological and affective characteristics of the process and provides new perspectives for this study of entrepreneurship education.

#### Practical implications

This study proposes entrepreneurial narrative as an effective means of entrepreneurship education. It stimulates the entrepreneurial passion of college students through the creative description of entrepreneurial events and then promote entrepreneurial intention. We explore the mechanism of entrepreneurial narrative on entrepreneurial intention and also provide a method for entrepreneurship education to solve the problem of effectiveness.

This paper has certain practical insights. First, colleges and universities need to build the foundation of entrepreneurial narrative, encourage faculty to use entrepreneurial narrative to teach knowledge, and invite entrepreneurs to tell the stories of their businesses. Furthermore, it is necessary to build detailed events and stories with entrepreneurial knowledge and to develop teachers’ ability to entrepreneurial narrative. College students are more likely to be inspired by positive entrepreneurial events and success stories [69]. It is recommended that entrepreneurial events and stories incorporate the entrepreneurial vision, mission, and values throughout the narrative. This allows college students to subconsciously understand, accept, and recognize the entrepreneurial spirit when receiving entrepreneurial narrative, thus triggering positive emotional resonance.

Second, entrepreneurial passion is an increasingly important factor in whether an individual will take the plunge into entrepreneurship. Passionate students are more likely to exhibit higher levels of creativity and persistence and put in greater effort toward entrepreneurship. Therefore, colleges and universities should consider the potential of entrepreneurial narrative to stimulate entrepreneurial passion, convey the expected image of entrepreneurship to college students through the construction of different discourse systems and the choice of rhetorical strategies, and provide them with the opportunity to identify with entrepreneurs.

Third, universities, governments, and companies should insist on providing entrepreneurial support. Sequentially entrepreneurial support not only helps college students to start their own businesses in terms of resources but also strengthens their confidence and motivates them to turn their passion and ideas into actions. This process can help students overcome the paradox of entrepreneurial identity encourage them to alter their career aspirations and stimulate entrepreneurial intention.

#### Limitations and future research

The research in this paper still has limitations. First, our study builds on affective events theory, which posits that the comprehension of events in a creative manner can facilitate the achievement of goals, accompanied by positive emotional outcomes. Entrepreneurial narrative that we focus on in this study is a creative language description of entrepreneurial stories and events. However, it is important to note that various modes of communication for entrepreneurial narrative, including language, video, lecture, and text, possess distinct presentation characteristics that can impact the audience differently [81,82]. Scholars have suggested that as well as being a tool for entrepreneurial success, narrative can also be a barrier to entrepreneurship [80]. Further research is required to explore in the various modes and characteristics of entrepreneurial narrative and their impact on entrepreneurial attitudes and activities.

Second, this paper examines the role of entrepreneurial passion in the relationship between entrepreneurial narrative and entrepreneurial intention from an emotional perspective. In the data collection process, we can only capture short-term entrepreneurial passion. Scholars have noted that short-term emotions may influence the entrepreneurs’ current state, but less so the entrepreneurs’ general entrepreneurial passion [27]. However, Gielnik (2015) pointed out that entrepreneurial passion is more flexible, and frequent short-term passions may also have a similar relationship with long-term passions [70]. Future research could adopt a more fine-grained perspective on the flexible patterns of entrepreneurial narrative, passion, and intention over time.

Third, we have examined the positive impact of entrepreneurial narrative on entrepreneurial intention. Previous research mostly concentrated on entrepreneurial intention assumes that all individuals have stable growth aspirations [4]. Future research could identify different patterns in the growth or decay of entrepreneurial intention across individuals over a period of time. Such research on fluctuations of intention would also add to our understanding of the dynamics inherent in motivational and emotional states.

## Supporting information

S1 Data (XLSX)
